# EDA-E7 Activated DCs Induces Cytotoxic T Lymphocyte Immune Responses against HPV Expressing Cervical Cancer in Human Setting

**DOI:** 10.3390/vaccines11020320

**Published:** 2023-01-31

**Authors:** Juan Feng, Yongliang Liu, Na Zhuang, Zixuan Chai, Limei Liu, Cheng Qian, Jiatao Li, Juanjuan Shan

**Affiliations:** Chongqing Key Laboratory of Translational Research for Cancer Metastasis and Individualized Treatment, Research Center for Precision Medicine, Chongqing University Cancer Hospital, Chongqing 400030, China

**Keywords:** cervical cancer, human papillomavirus (HPV), extra domain A (EDA), cancer immunotherapy

## Abstract

Cervical cancer is a major cause of cancer death in women worldwide. Targeting human papillomavirus (HPV) viral oncoproteins E6 and E7 is a new strategy for cervical cancer immunotherapy and has been associated with resolution of HPV-induced lesions. How to efficiently induce T cell target killing of HPV infected cervical cancer is of great potential benefit for cervical cancer treatment. Fusion protein containing the extra domain A (EDA) from fibronectin, a natural ligand for Toll-like receptor 4 (TLR4), and HPVE7 (EDA-E7) has been shown to efficiently induce dendritic cells maturation and trigger specific antitumor CD8+ T cells response in mice. In this study, we constructed EDA-E7 fusion protein of human origin and tested its function in dendritic cell maturation as well as antitumor T cell response. We found that EDA-E7 could be efficiently captured by human PBMC derived dendritic cells (DCs) in vitro and induce DCs maturation. Importantly, this effect could work in synergy with the TLR ligand anti-CD40 agonist, polyinosinic-polycytidylic acid [poly (I:C)], R848, and CpG2216. EDA-E7 matured DCs could activate T cells and trigger an anti-tumor response in vitro. Single cell RNA sequencing and T cell targeted killing assay confirmed the activation of T cells by EDA-E7 matured DCs. Therefore, therapeutic vaccination with EDA-E7 fusion protein maybe effective for human cervical carcinoma treatment.

## 1. Introduction

Cervical cancer is a major cause of cancer death in women worldwide. Consistent evidence has indicated that human papillomavirus infection (HPV) is the main factor that induces cervical lesions and cervical cancer [1]. It is worth noting that while the detection rate of HPV in cervical cancer tissue is as high as 99%, the HPV genotypes 16 and 18 are 73.8% and 16.4% in Southwest of China, respectively [2,3]. Traditional treatment methods such as surgery, radiotherapy, chemotherapy, etc., are still the preferred treatment at present for cervical cancer. However, the effect is not satisfactory for advanced stage, metastasis, and recurrent cervical cancer [4,5]. The HPV viral oncoproteins E6 and E7 are considered tumor-specific targets for immunotherapy, making them as the most effective vaccine for cervical cancer treatment.

With the development of molecular cell biology and immunology, basic study and clinical trials have been carried out to evaluate the efficiency and safety of immunotherapy in cervical cancer. In 2018, the National Comprehensive Cancer Network (NCCN) recommended Pembrolizumab, one of the immune checkpoint inhibitors targeting PD-1, as a new treatment for unsatisfactory advanced, metastatic, and recurrent cervical cancer [6]. However, the inhibitor is effective only for patients with high expression of PD-L1, while has no benefit for patients who do not express or express low level of PD-L1 [7]. Therefore, our attention was back on a tumor vaccine to stimulate HPV antigen specific T cells for the treatment of cervical cancer. Ferrara et al. reported a clinical trial for autologous DCs stimulated by recombinant HPV16E7 or HPV18E7 protein in the treatment of patients with advanced, metastatic, and recurrent cervical cancer, and found that the recombinant HPV E7 induced antitumor T cell responses in a portion of late stage cervical cancer patients [8].

Due to their outstanding antigen presenting ability, DCs are the key mediator of T cell immune response. They capture and process antigens in the context of major histocompatibility complex (MHC) to naïve T cells, and trigger a specific adaptive immune response. However, the ex vivo DCs-based vaccine is difficult to standardize, therefore, in vivo induction of DCs with HPV antigen as vaccine for cervical cancer has great potential for clinical application. It was reported that the spliced exon encoding the type III repeat extra domain A (EDA) from fibronectin, which is produced in response to tissue injury and works as a damage-associated molecular pattern molecule [9], is able to target antigens to DCs while inducing maturation through TLR4 ligation [10,11,12,13]. Moreover, scientists have amplified mouse origin EDA and constructed recombinant fusion protein EDA-E7 (HPV16E7), then evaluated the immune response in mouse condition and found that EDA-E7 could efficiently induce specific immune rejection of HPV16E7 infected TC-1 tumors [14]. In the present work, we cloned human origin EDA and successfully constructed and purified EDA-HPV16E7 (EDA-E7), a fusion protein containing human EDA and part of HPV16E7. Further, EDA-E7 and Toll-like receptor (TLR) agonist were applied to induce human DCs cell maturation in vitro. Specific activation of human T cells by EDA-E7 matured DCs, and T cells mediated cell lysis of HPV16E7 infected cervical cancer cell were observed. Our research will fill the gap between bench study and clinical application in human for the treatment of HPV infected cervical cancer using EDA-E7 vaccine.

## 2. Materials and Methods

### 2.1. Cell Culture

SiHa, 293(HEK-293) and THP-1 were from Shanghai Cell Collection (Shanghai, China). SiHa and 293 were cultured in DMEM (Gibco, Beijing, China) and supplemented with 10% fetal bovine serum (FBS) and penicillin/streptomycin (Gibco, Beijing, China). THP-1 were cultured in RPMI 1640 (Gibco, Beijing, China) supplemented with 10% fetal bovine serum (FBS) and penicillin/streptomycin.

DCs culture: buffy coat is from healthy donors as approved by the ethics committee of Chongqing University Cancer Hospital. A total of 15 donors were used in this study. Each experiment was biologically replicated at least. Human PBMC were purified from buffy coat by density gradient centrifugation using Ficoll (Cytiva, Grens, Switzerland). PBMC were suspended in RPMI 1640 (Gibco, Beijing, China) basic medium with concentration of 2 × 10^6^/mL. Seed PBMC in 24 well plate and culture in 37 °C, 5%CO_2_ for 90 min. To obtain DCs, the attached mononuclear cells were cultured for additional 5 days in RPMI 1640 (Gibco, Beijing, China) supplemented with 5% human serum, 100 U/mL penicillin and 100 µg/mL streptomycin (P/S), 2 mM L-glutamine, 800 IU/mL GM-CSF (Peprotech, Cranbury, NJ, USA) and 200 U/mL IL-4 (Peprotech, Cranbury, NJ, USA). For DCs maturation, 500 nM EDA-E7, 500 nM EDA-E7 + 10 ug/mL poly (I:C) (Invivogen, Toulouse, France), 500 nM EDA-E7 + 2 uM CPG (CPG2216, Invivogen, Toulouse, France), 500 nM EDA-E7 + 1 ug/mL R848 (Invivogen, Toulouse, France), or 500 nM EDA-E7 + 100 ng/mL anti-CD40 (Abcam, Cambridge, UK) were added into DCs culture medium on day 6 after DC stimulation.

For lymphocyte purification, we collected the unattached cell after incubating the PBMC in a 24 well plate for 90 min as described above, then purified T cells using human Pan T Cell Isolation Kit (Miltenyi Biotec, Gaithersburg, MD, USA) and cultured them in lymphocyte serum-free medium (Dayou, cat#:6111021, Hangzhou, China). For T cell activation, on day 7 of DCs culture, 1:1 (DCs:T cells) naïve pan T cells were added into DCs cells with addition of 200 U/mL IL-2, 30 ng/mL IL-21, 5 ng/mL IL-15. and 5 ng/mL IL7, then cultured in 37 °C, 5%CO_2_ for 10 days before analysis.

### 2.2. Human Recombinant EDA-E7 Fusion Protein Preparation

RNA from SiHa was isolated with RNA isolation kit (Omega, Atlanta, GA, USA) and reverse transcript into cDNA using PrimeScript RT reagent kit from Takara. We depleted pRB binding domain of HPVE7, then link E7 1-29 first amino acids (aa) to the N terminal of EDA and 43-98 aa to the C terminus of EDA to make target recombinant protein sequence: E7(1-29)aa + EDA + E7(43-98)aa (EDA + E7), while control protein without EDA as: E7(1-29)aa + E7(43-98)aa (E7). To construct the fusion protein, we used the over-lap PCR method. PCR primers are shown in Table 1.

**Figure 1:**
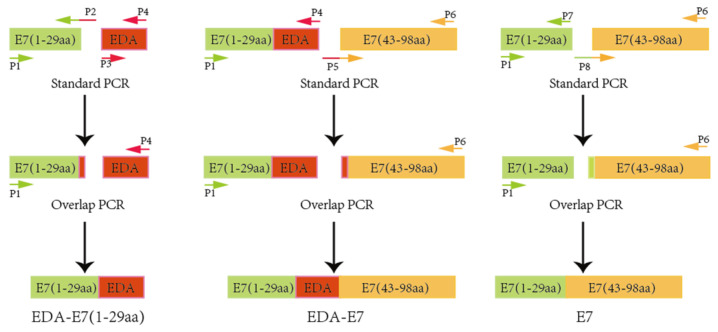
Schematic diagram for EDA-E7 and E7 PCR strategy.

To construct E7(1-29)aa + EDA target DNA, primers 1 and 2 were used and SiHa cDNA was applied as a template to amplify E7(1-29)aa target sequence; while primer 3 and 4 were used and 293 cells cDNA were used as template to amplify human EDA target sequence. Purify E7(1-29)aa and EDA target DNA and link them to get E7(1-29)aa + EDA target DNA. E7(1-29)aa + EDA from previous step were further amplified using primer 1 and 4. E7(43-98)aa were amplified with primer 5 and 6, then linked to E7(1-29)aa + EDA. To get E7(1-29)aa + E7(43-98)aa DNA, product of primer 1 and 7 with template of SiHa cDNA, and product of primer 6 and 8 with template of EDA expression plasmid were linked together via the overlap sequence. For the construction of pET20b expressing EDA-E7 or E7, plasmid pET20b (Kindly provided by Prof. Jesu´s Prieto from Centro de Investigacio´n Me´dica Aplicada CIMA, Pamplona, Spain) and target DNA were digested with restriction enzyme NdeI and NotI, then ligated to get pET20b-EDA-E7 and pET20b-E7. Cloning protocol is outlined as below (Figure 1):

To obtain EDA-E7 or E7 recombinant protein, pET20b-EDA-HPVE7 and pET20b-E7 were transfected into BL21 (DE3) E-coli; incubate in 37 °C, 260 r/min shaking culture. Add 0.5 mM IPTG (isopropylthio-β-galactoside, purchased from Thermo Fisher Scientific, Shanghai, China) to the culture when the BL21 OD600 reached 0.5, then culture another 4h before protein purification. Affinity chromatography were applied to purify EDA-E7 and E7 recombinant protein. Then, elute the protein with different concentration of imidazole solution. SDS-PAGE and Coomassie brilliant blue staining were applied to confirm the target protein size as well as purity. Recombinant protein was purified with affinity chromatography as described previously [14]. As the recombinant protein exists mainly in the inclusion body, protein renaturation using gentle removal of urea was applied as described previously [15].

### 2.3. Analysis of E7, EDA-E7 Binding with DCs

E7 and EDA-E7 were labeled with LinKine™ FITC Labeling Kit (Abbkine, Wuhan, China), then 100 nM E7 or EDA-E7 was added into DCs culture. DCs binding with E7 or EDA-E7 was measured through FITC signal by a fluorescent microscope and flow cytometry after 24 h of incubation.

### 2.4. Flow Cytometry

For surface staining, cells were blocked with 2% normal rabbit serum and subsequently stained with fluorochrome-conjugated antibodies in FACS buffer (PBS + 2%FBS + P/S) at 4 °C for 30 min and analyzed with cytometer (Beckman coulter cytoflex). DCs activation were analyzed using anti-HLA-DR (Biolegend, San Diego, CA, USA), anti –HLA-ABC (BD Biosciences, New Jersey, USA), anti-CD80 (Biolegend, San Diego, CA, USA), anti-CD83 (Biolegend, San Diego, CA, USA), and anti-CD86 (Biolegend, San Diego, CA, USA). For T cells activation analysis, DC activated T cells were collected by centrifugation of the suspended pan T cells in 1000 rpm for 5 min, then stained with anti-CD3, anti-CD4, anti-CD8, anti-CD107a, anti-4-1BB, and anti-OX40 (Biolegend, San Diego, CA, USA) for flow analysis.

Intracellular staining for DCs IL12, TNFα: activated DCs were washed, fixed, and permeabilized using BD Cytofix/Cytoperm kit (BD Bioscience, Franklin Lakes, NJ, USA) at 4 °C for 20 min. The cells were then stained with anti-IL-12 and anti-TNFα (Biolegend, San Diego, CA, USA) in permeabilization solution following the protocol provided. Data was acquired on Beckman coulter cytoflex.

For the analysis of TLR7 and TLR9 expression on DCs, DCs were fixed and permeabilized as described above. Then, stained with anti-TLR7 and anti-TLR9 (Biolegend, San Diego, CA, USA) for flow analysis.

For caspase-3 staining, target cells SiHa were washed after coculture with T cells for 4 h to remove T cells, then digested to single cells with trypsin (Gibco, Beijing, China). The cells were fixed and permeabilized as described previously using BD cytofix/Cytoperm kit, and stained with anti-cleaved caspase-3 (BD Bioscience, Franklin Lakes, NJ, USA) before analysis.

### 2.5. Monocyte Activation Analysis

To analyze the function of TLR4 for EDA-E7 induced NF-KB activation, EDA-E7 was added to the THP-1 cells with or without addition of TLR4 blocking antibody (Biolegend, cat#: 312802, 20 μg/mL, San Diego, CA, USA). After incubation for 10 min, intracellular flow cytometry for P-p65 was performed to analyze the phosphorylation of p-65. Briefly, stimulated THP-1 cells were fixed and permeabilized as previously described, then stained with anti-P-p65 (CST, cat#: 3033S, Danvers, MA, USA) for 30 min at 4 °C. Washed and stained with secondary antibody, the anti-rabbit conjugated with Alexa fluor 647, then washed and analyzed with flow cytometer.

To determine which time point could achieve the best effect of THP-1 activation, THP-1 cells were cultured in a 12 well plate and 100 nM EDA-E7 were added to the culture. After coculture for 0 min, 5 min, 10 min, we collected the cells for analysis of p65 phosphorylation with Western blot. To test the dose effect of EDA-E7 on TLR4 pathway activation, THP-1 were treated with different doses of EDA-E7 and cultured in 37 °C, 5% CO_2_ for 10 min. Cells were collected and washed with PBS, then lysed with RIPA buffer supplemented with protease and phosphatase inhibitor cocktail (Roche, Switzerland). Purified and degenerated proteins were loaded onto 12% SDS-PAGE gels followed by electrophoretic transfer to nitrocellulose membranes. Primary antibodies for p65 and P-p65 were purchased from Abcam (Cambridge, UK); anti-β-actin antibody was from CST company (Danvers, MA, USA).

### 2.6. T Cell Proliferation Assay

For T cell proliferation, matured DCs (5 × 10^5^) induced by different combinations of TLR agonists were cocultured with Naive Pan T (1:1) in 6-well plates in DCs culture medium. Cells were counted on day 7 and day 10 post coculture. There are 3 replicates for each group and the proliferation rate of each group was calculated based on the mean total cell number for each group. Since DCs could hardly proliferate during in vitro culture, it is difficult to separate T cells from DCs for cell counting. In addition, on day 7 and day 10 post coculture, total cell number of DCs and T cells reached more than 10 million. Therefore, we considered that the number of DCs would not affect the growth curve of T cells.

### 2.7. T Cell Receptor (TCR) Coupled Single Cell RNA Sequencing

Collect T cells on day7 of T cells activation by EDA-E7 matured DCs for TCR coupled single cell RNA sequencing. Sequencing was completed by Beijing Genomics Institute (Beijing, China). To identify clonotypes, we used a 10×Genomics Cell Ranger pipeline with alignment and annotation according to the manufacturer’s instruction. TCR were aligned to GRCh38 reference genome. In-frame TCR alpha-beta pairs were considered as dominant TCR of a single cell.

### 2.8. T Cell In Vitro Cytotoxicity Assay

To overexpress HLA-A*02:01 in SiHa, HLA-A*02:01 expression sequence was synthesized in Gene Create company and constructed into a lentiviral plasmid with GFP as a transfection reporter gene. SiHa transfected with HLA-A*02:01 expressing lentivirus were sorted by GFP reporter using flow cytometry. 

HPV16E7 11–19 episode specific TCR cloning and targeted cytotoxic assay were conducted accordingly to a previously published article [16]. Briefly, PBMC derived T cells were activated using CD3 and CD28 activation beads. TCR overexpression lentivirus was added into the activated T cells at 36h post activation. Percentage of TCR expression T cells were confirmed by flow cytometry for reporter GFP expression. We could get around 80% GFP + T cells from each infection, which we consider sufficient for in vitro experiment. On day 7 post infection, TCR expression T cells were incubated with wild type SiHa or HLA-A*02:01 expressing SiHa with E:T ratio 5:1 in SiHa culture medium (DMEM + 10%FBS + P/S). As SiHa were labeled with luciferase, we used luciferase signal to measure the lysis efficiency of T cells. After incubation for 12 h, luciferase assay was conducted to evaluate T cell cytotoxicity.

For EDA-E7 induced T cell specific cytotoxicity assay, control E7 or EDA-E7 matured DCs were incubated with PBMC derived CD8+ T cells with ratio DCs:T as 1:4 to activate T cells. After incubation for 7 days, DCs activated T cells were purified with magnetics beads for 4-1BB. Then, 1 × 10^4^ HLA-A*02:01 expressing and wild type SiHa were seeded in 96 well plate, after attachment overnight, and T cells were added into SiHa with indicated ratios. T cell lysis efficiency was analyzed by luciferase signal at 12 h post incubation. For apoptotic assay, T cells activated by E7 or EDA-E7 stimulated DCs were cocultured with HLA-A*02:01 expressing or wild type SiHa with E:T ratios 5:1 and 10:1 for 4 h before flow cytometry for caspase 3.

### 2.9. Statistical Analysis

Data is presented as mean ± SD. Statistical comparisons between groups were analyzed by a student’s *t*-test. A *p* value < 0.05 was considered statistically significant.

## 3. Results

### 3.1. Recombinant Fusion Protein EDA-E7 Stimulates DCs Maturation through TLR4 Signaling Pathway

**Figure 2:**
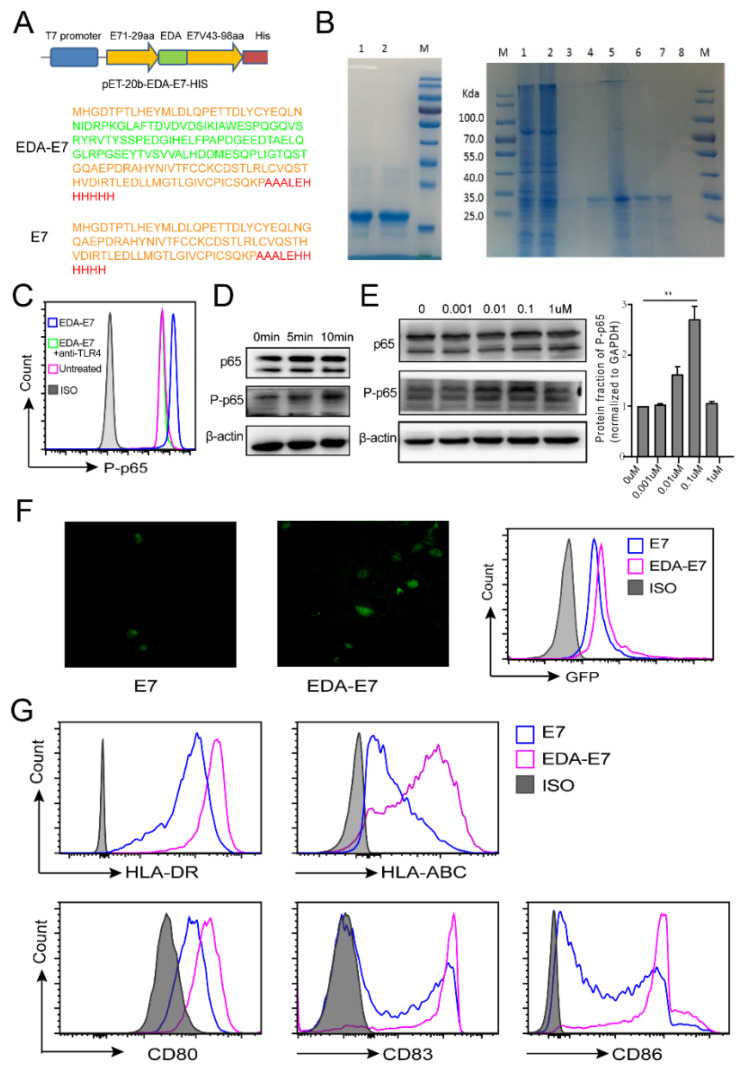
Recombinant fusion protein EDA-E7 activates DCs through TLR4. Schematic diagram showing pET-20b-EDA-E7-His plasmid design as well as protein sequence of fusion protein EDA-E7 and E7 (**A**); SDS-PAGE Coomassie brilliant blue staining for E7and EDA-E7. Left lane 1 and 2: E7 recombinant protein after purification, protein marker size is as right panels. Right lane 1: sample before purification, lane 2: sample of flow-through, lane 3: 1% imidazole elution, lane 4: 2% imidazole elution, lane 5: 5% imidazole elution, lane 6: 50% imidazole elution, lane 7: 100% imidazole elution, lane 8: elution control (**B**); flowcytometry for P-p65 with or without TLR4 blockade after EDA-E7 treatment (**C**);Western blotting for p65, P-p65 at different time point after THP-1 treatment of 100nM EDA-E7 or E7 (**D**); Western blotting for p65, P-p65 upon stimulation with different dose of EDA-E7 protein and the quantification graph (n = 3 per group) (**E**); Fluorescent image and flow cytometry for E7, EDA-E7 binding with DCs (**F**); Flow cytometry for HLA-DR HLA-ABC, CD80, CD83, CD86 expression on DCs after stimulation with EDA-E7 or E7 (**G**), ISO: isotype control,** *p* < 0.01.

EDA from fibronectin could activate a TLR-4 signaling pathway of dendritic cells (DCs) and fusion protein EDA-OVA, EDA-E7 could stimulate specific CTL killing of OVA expression tumor cells and HPV-E7 infected tumor cells, respectively, in a mouse [11]. Nevertheless, whether EDA-E7 could be used for activation of DCs and trigger antigen specific cytotoxic T lymphocyte (CTL), a killing in human setting is not known. To address this question, we amplified human EDA from human 293 cells [14], and constructed recombinant fusion protein of EDA-HPV16E7 (EDA-E7) expressing plasmid with his tag as shown in Figure 2A. The protein sequence of EDA-E7 and control E7 was also demonstrated accordingly in Figure 2A. EDA-E7 and control protein E7 were expressed and purified using affinity chromatography. Protein purity was confirmed by SDS-PAGE stained with Coomassie brilliant blue (Figure 2B).

In mouse conditions, it was reported that EDA-E7 activates dendritic cells through TLR4. As human monocyte cell line THP-1 cells express TLR4, we checked whether the EDA-E7 recombinant protein obtained in our system could activate THP-1 through TLR4 signaling pathway. TLR4 activation leads to phosphorylation of p65, one component of the NF-KB complex [17]. We used flowcytometry for THP-1 P-p65 with or without TLR4 blockade after EDA-E7 stimulation. From Figure 2C, EDA-E7 stimulation significantly increased the phosphorylation of p-65, however, after a blockade of the function of TLR4 with TLR4 blocking antibody, THP-1 phosphorylation level of p-65 dropped to basal level. This data demonstrated that EDA-E7 stimulates NF-KB pathway through TLR4. We then asked which time point could achieve the best activation of TLR4 signaling pathway by EDA-E7, and we analyzed p65 phosphorylation within 10 min after stimulation with EDA-E7 concentration of 100 nM. We choose 100 nM as starting concentration because it was reported in mouse condition that EDA-E7 100 nM could achieve best activation of mouse DCs [11]. From Figure 2D we could see that best phosphorylation of p65 was at 10 min post stimulation. Therefore, we used 10 min stimulation as the time point for the analysis of which dose of EDA-E7 could achieve best effect on TLR4 signaling pathway activation. From Figure 2E we found that p65 phosphorylation was upregulated upon EDA-E7 treatment in a dose dependent manner from 0 uM to 0.1 uM.

Previous experiments only showed that EDA-E7 could activate THP-1 TLR4 signaling pathway. We decided to study whether EDA-E7 could activate human monocyte derived DCs. We firstly checked whether EDA-E7 recombinant protein could be captured by DCs. E7 and EDA-E7 were labeled with LinKine™ FITC Labeling Kit (Abbkine, Wuhan, China), then 100 nM E7 or EDA-E7 were added to human monocyte derived DCs. After coculture for 24 h, FITC positive DCs were observed in both E7 and EDA-E7 treated group as shown in Figure 2F. Flow cytometry indicated that the binding efficiency of EDA-E7 to DCs may be higher than E7 alone, but this may also be because of higher labeling efficiency of FITC on EDA-E7 than E7 (Figure 2F). Maturation of DCs upregulates the expression of cell surface MHC genes, co-stimulatory molecules as well as pro-inflammatory cytokines such as TNFα and IL12 [18]. Therefore, to demonstrate the role of EDA-E7 on DCs activation, we evaluated the activation marker of DCs with flow cytometry for HLA-DR, HLA-ABC as well as co-stimulatory molecules CD80, CD83, and CD86. The results showed that EDA-E7 upregulated both the MHC proteins and the costimulatory molecules with much higher efficiency than E7 alone (Figure 2G).

### 3.2. 500 nM EDA-E7 Has the Best Effect to Activate DCs In Vitro

**Figure 3:**
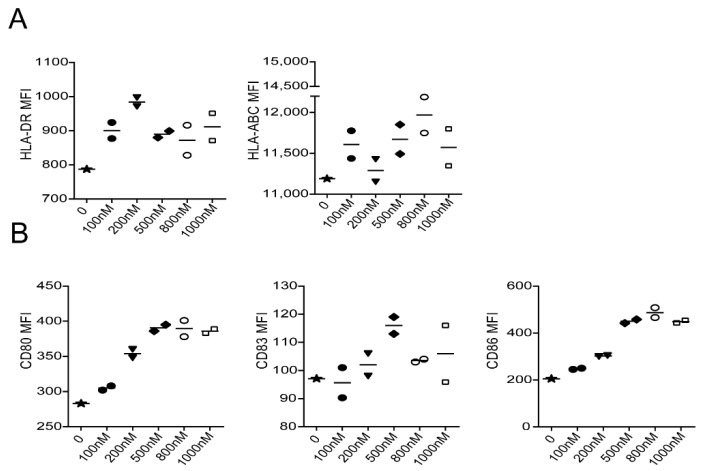
EDA-E7 concentration trial for DCs activation. Flow cytometry for antigen presenting molecular expression on DCs after stimulation with different dose of EDA-E7 (**A**); flow cytometry for costimulation molecule expression on DCs after EDA-E7 stimulation (**B**). n = 2 per group, data was presented as mean, MFI: mean flow index.

Previous data in THP-1 cell showed a dose dependent manner for EDA-E7 on TLR4 pathway activation from 0 uM to 0.1 uM, we asked which dose of EDA-E7 works more efficiently for DCs activation in vitro. We tried 100 nM, 200 nM, 500 nM, 800 nM, and 1000 nM for DCs activation and used flow cytometry for MHC molecule and costimulatory molecule as activation marker. Even though DCs were almost 100% positive for all these markers compared to unstained control, mean flow index data showed that from 0 nM to 500 nM, activation effect of EDA-E7 on DCs increased with dosage. However, after 500 nM, higher concentration of EDA-E7 did not achieve better activation (Figure 3). We conclude that 500 nM EDA-E7 is the best concentration for DCs activation in vitro. Therefore, we used 500 nM EDA-E7 in this study for other function analysis unless otherwise mentioned.

### 3.3. TLR Activators Upregulated the Activation Effect of EDA-E7 on DCs

**Figure 4:**
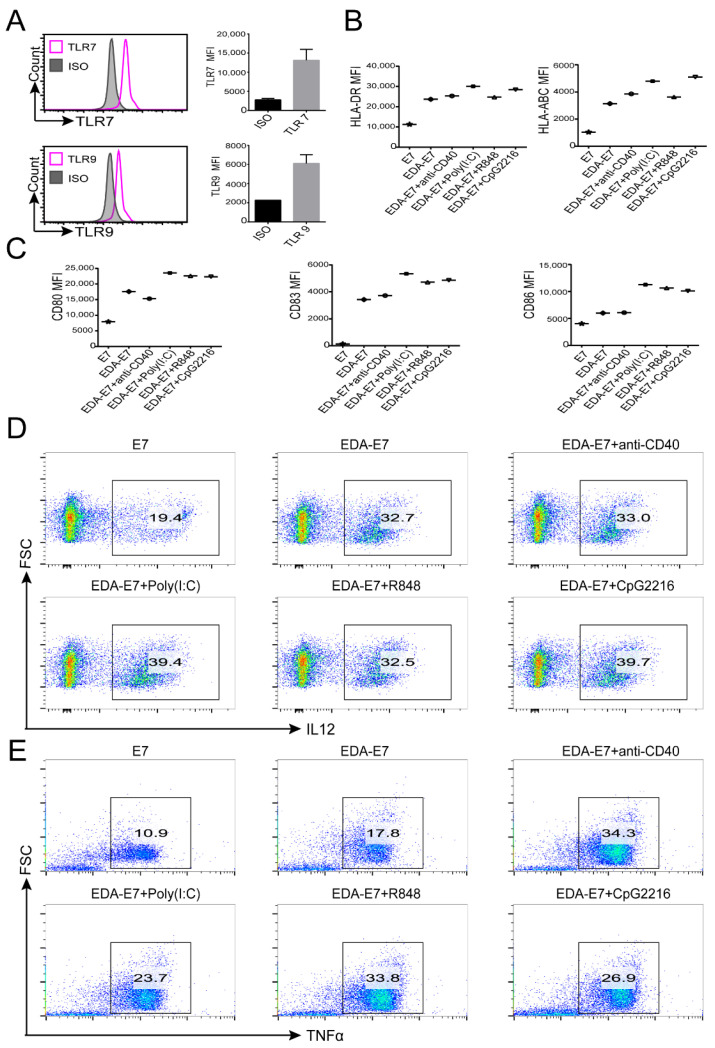
TLR activator upregulated EDA-E7 effect on DCs activation. Flow cytometry analysis for TLR7 and TLR9 expression on monocyte derived DCs (MFI: mean flow index, ISO: isotype control) (**A**); Flow cytometry for DCs antigen presenting molecule expression after stimulation with EDA-E7 and TLR activators (**B**) (n = 2 per group, data was presented as mean); Flow cytometry for DCs costimulatory molecule after stimulation with EDA-E7 and TLR activators (**C**); Flow cytometry for DCs pro-inflammatory cytokines IL-12 (**D**) and TNF α after stimulation (**E**).

Previous studies have shown that TLR activators anti-CD40 agonist, poly (I:C) (binds TLR3), R848 (binds TLR7/8), and CpG2216 (binds TLR9) has the potential to stimulate the activation of DCs [14]. We asked whether combined use of anti-CD40 agonist, poly (I:C), R848 and CpG2216 with EDA-E7 would work in synergy to stimulate DCs maturation. We firstly checked the expression of TLR7 and TLR9 because contrary results have been reported about their expression on human monocyte derived DCs. From Figure 4A, we found that both TLR7 and TLR9 were expressed on human monocyte derived DCs. Then, we treated DCs with EDA-E7, EDA-E7 + anti-CD40, EDA-E7 + poly (I:C), EDA-E7 + R848, EDA-E7 + CpG2216, and E7 only as control. Flow cytometry was applied to evaluate the activation markers of DCs including antigen presenting molecule as well as costimulatory molecules. From the results, we found that EDA-E7 + anti-CD40 did not show a better effect compared with EDA-E7, however, TLR3 activator poly (I:C), TLR7/8 activator R848 and TLR9 activator CpG2216 indeed upregulated the efficiency of EDA-E7 to stimulate DCs (Figure 4B,C). In addition to antigen presenting molecules and costimulatory molecules, we also checked the expression of pro-inflammatory cytokine IL-12 and TNFα in the DCs with flow cytometry. In accordance with the surface markers, IL12 and TNFα expression by DCs were also elevated after EDA-E7 stimulation. Importantly, this effect was further upregulated by combination use of poly (I:C), E7 + R848 or CpG2216. Interestingly, when combined use of EDA-E7 with anti-CD40 agonist, TNFα expression level was also elevated even though no significant upregulation for IL12, MHC molecules or costimulatory pathway molecules (Figure 4D,E).

### 3.4. EDA-E7 Matured DCs Could Activate T Cells In Vitro

**Figure 5:**
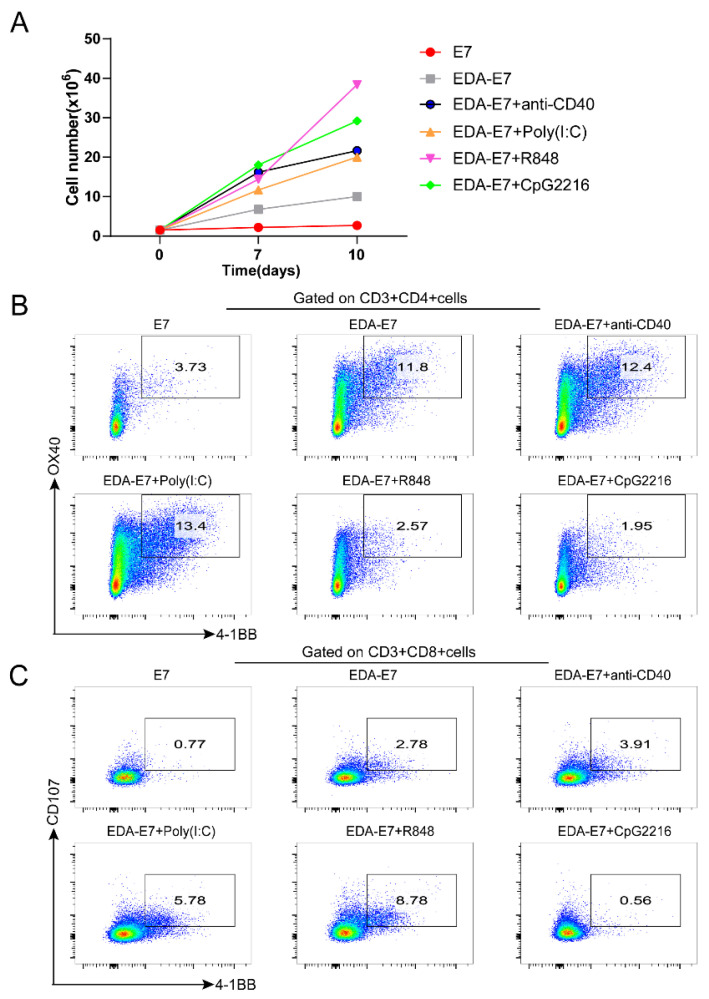
Combined use of EDA-E7 with TLR agonist upregulated the activation of CD4 + T and CD8+ T cells by DCs compared to EDA-E7 alone or E7. T cells growth curve after coculture with DCs, n = 3 per group (**A**); Flow cytometry for activation markers of CD4 + T cells (**B**); Flow cytometry for activation markers of CD8+ T cells (**C**).

As a specialized antigen-presenting cell, DCs are the key mediator of T cell immune response. They capture, ingest, and process related antigens, then present the antigen to naive T cells and trigger a specific immune response. We thus asked whether EDA-E7 stimulated DCs could activate T cells in vitro. We treated DCs with E7, EDA-E7 or EDA-E7 in combination with anti-CD40, poly (I:C), E7+R848, CpG2216, then co-culture the DCs with human PBMC derived T cells. OX40 and 4-1BB were used as CD4+T cell activation markers, while CD107 and 4-1BB were used as CD8+ T cell activation markers as reported previously [19]. We firstly analyzed the proliferation of T cells, which could indicate the activation of T cells. We found that even though we seeded the same number of naïve T cells before activation, T cell number increased after co-culture with EDA-E7 stimulated DCs compared with E7 stimulated DCs on day 10 post activation. Moreover, combined use of EDA-E7 with TLR activators increased the proliferation of T cells compared to EDA-E7 used alone (Figure 5A). On day 10 post activation, we collected the T cells for the analysis of T cell activation markers. From flow cytometry, we found that compared to E7 stimulated DCs, activation efficiency of EDA-E7 matured DCs on CD4 + T cells and CD8+ T cells was both upregulated (from 3.73% to11.8 for CD4 + T cells and from 0.77% to 2.78% for CD8+ T cells). Poly (I:C) treatment further increased CD4 + T cells activation percentage from 11.8% to 13.4%; while anti-CD40, poly(I:C) and R848 treatment increased CD8+ T cells activation percentage from 2.78% to 3.91, 5.78, and 8.78, respectively. These data indicated that EDA-E7 treated DCs activate T cells more efficiently than E7, and combination of EDA-E7 with the TLR3 ligand poly (I:C), which promotes T cells proliferation and survival through the production of type I IFN [20,21] and has the best efficiency to further improve T cell activation (Figure 5B,C).

### 3.5. TCR Coupled Single Cell RNA Sequencing Revealed TCR Enrichment and Cytotoxic Property of T Cells after Co-Culture with EDA-E7 Activated DCs

**Figure 6:**
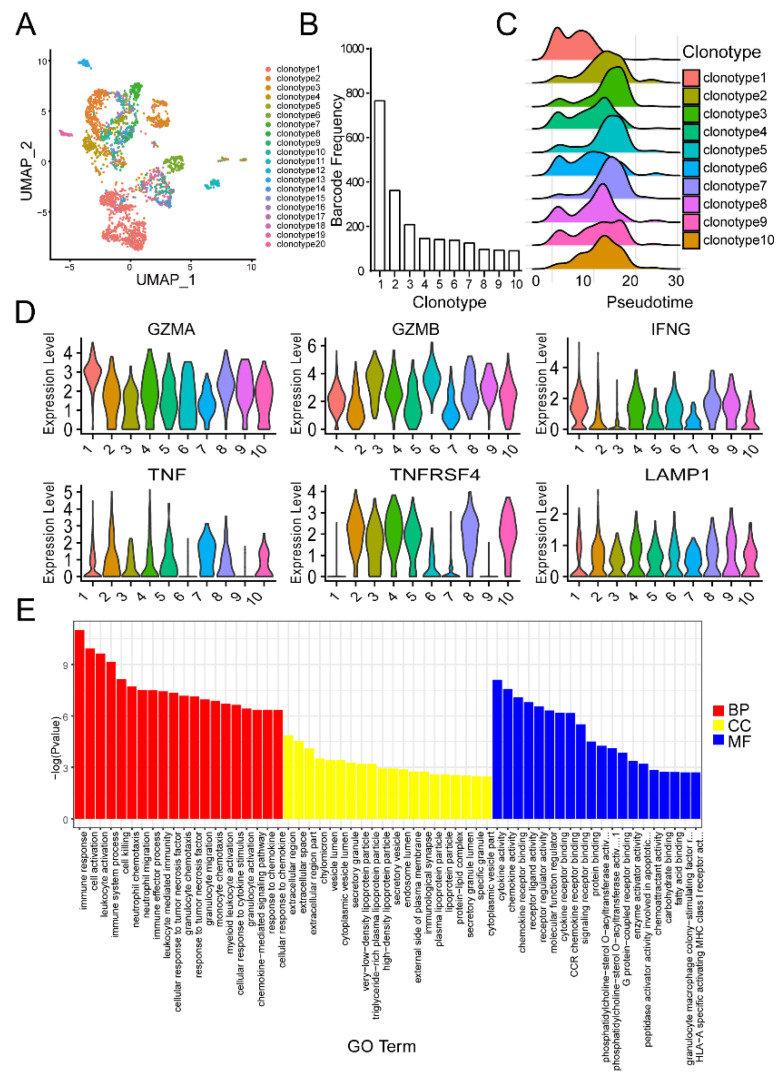
TCR coupled single cell RNA sequencing for DCs activated T cells. UMAP for TCR coupled single cell RNA sequencing showing top 20 clonotypes after activation by DCs (**A**); graph for top 10 clonotypes frequency (**B**); pseudotime analysis for top 10 clonotypes (**C**); representative inflammatory cytokines expression in top 10 clonotypes (**D**); GO analysis showing the enriched pathways in biological process (BP), cellular component (CC) and molecular function (MF) (**E**).

To determine whether there is clonal selection and amplification of T cells after DCs stimulation, we analyzed the results from TCR coupled single cell RNA sequencing for total T cells stimulated with EDA-E7 matured DCs. From Figure 6A, we could see that each cluster was composed of different combinatorial subsets of clonotypes. Clonal expansion was observed with clonal sizes ranging from 1 to 765 (Figure 6B). CD8+ T cells had more clonal cells than CD4 + T cells. Naïve CD4 + T cells and CD8+ T cells displayed very limited clonal expansion. Cytotoxic clonetype1, which expressed high level of granzyme A, B, in IFNG et al., showed higher expansion than other clonotypes (Figure 6B). Pseudotime analysis indicated clonotype1 emerged as the earliest T cell clonotype activated by DCs (Figure 6C). The top 10 frequently expanded clonotypes all showed high expression of GZMA, GZMB, IFNG, TNF, LAMP1 (Figure 6D). To analyze the function of clonotypes, we used GO enrichment analysis to identify pathways that have been enriched in the T cells after stimulation. The results indicated biological process (BP), especially immune response related pathway, were enriched in the activated T cells. For cellular components (CC) analysis, we found extracellular components ranked most significantly upregulated, indicating immune related cytokines may be elevated in the activated T cells. Molecular function (MF) analysis found that cytokines activity was upregulated most significantly, which was in accordance with BP and CC results (Figure 6E).

### 3.6. T Cells Activated by EDA-E7 Matured DCs Efficiently Kills HPV16E7 Infected SiHa

**Figure 7:**
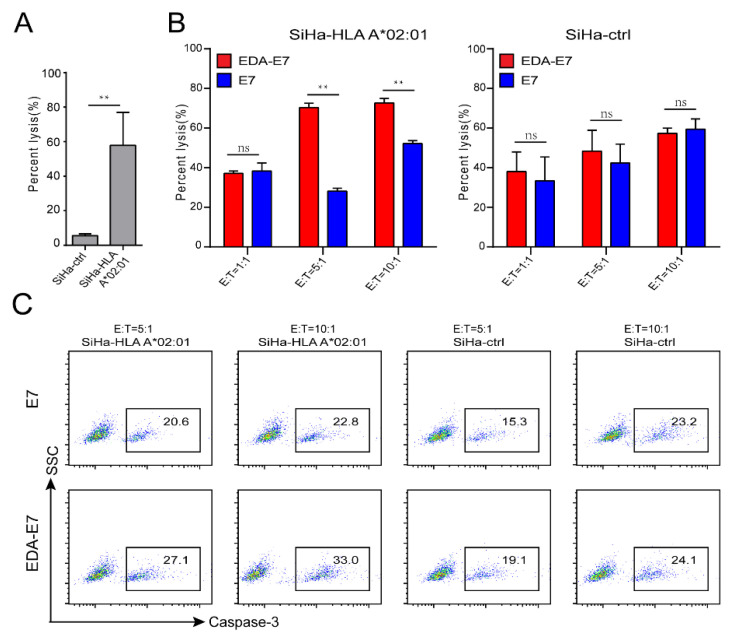
Targeted killing of HPV infected SiHa cells by the activated T cells. TCRT specific lysis assay confirmed the function of HLA-A*02:01 in SiHa (**A**); luciferase lysis assay for T cell target killing of wild type and HLA-A*02:01 expressing SiHa cells at 12 h with different E:T ratios (**B**); cleaved caspase3 flow cytometry for SiHa cells after co-culture T cells (**C**), ** *p* < 0.01, ns: no significance.

Since we have shown that EDA-E7 could stimulate DCs, and DCs would present E7 antigen to activate naive T cells. We further asked whether the activated T cells could specifically target HPV16E7 infected cancer cells. HLA-A*02:01 is relatively common in the Chinese population and we confirmed that HLA-A*02:01 indeed is the main subtype of HLA in PBMC cells, thus, making sure T cell cytotoxicity against SiHa was HLA restricted antigen specific, we overexpressed HLA-A*02:01 in SiHa cells. As HLA-A*02:01 expressing plasmid contain GFP reporter, we could use flow cytometry for GFP to sort HLA-A*02:01 expressing SiHa cells. To test the function of HLA-A*02:01 on SiHa, we constructed TCRT cells with the expression of a reported TCR, which could specifically target HPV16 E7 11–19 episode [16]. From Figure 7A, we found that in vitro activated T cells, which express E7 specific TCR, could lyse wild type SiHa with low efficiency (lower than 10% at 12 h post incubation), but efficiently target HLA-A*02:01 expressing SiHa (efficiency about 60% at 12 h post incubation) (Figure 7A). This data demonstrated that HLA-A*02:01 expression in SiHa was successful and functional.

After confirmation of the function of HLA-A*02:01 on SiHa target cells, we tested the effect of EDA-E7 on T cell cytotoxicity. E7 or EDA-E7 stimulated DCs were co-cultured with naïve T cells, then activated T cells were purified using magnetic beads for T activation marker 4-1BB. Activated T cells were co-cultured with SiHa cells with or without expression of HLA-A*02:01. We tried effector T cells to target cell ratio (E:T) as 1:1, 5:1, and 10:1, and analyzed the lysis efficiency at 12 h post co-culture. As target cells SiHa were labeled with luciferase, we could use luciferase signal to determine the lysis percentage. From Figure 7B we could see that EDA-E7 stimulated T cells have significantly better lysis efficiency on SiHa-HLA-A*02:01 compared to E7 control group or on wild type SiHa for all E:T ratio groups (EDA-E7 on SiHa-HLA-A*02:01:75%; E7 on SiHa-HLA-A*02:01:35%; EDA-E7 on wild type SiHa: 40% at E:T = 5:1). Importantly there was no difference regarding the lysis efficiency between EDA-E7 and E7 group for wild type SiHa, supported that T cells activated by EDA-E7 stimulated DCs could achieve antigen specific cytotoxicity against SiHa-HLA-A*02:01 (Figure 7B). We then used flow cytometry for caspase-3, which is the marker for apoptotic cells to further confirm the cytotoxicity of the T cells. We chose E:T ratios 5:1 and 10:1 for the apoptosis assay. Consistently, we found that T cells activated by EDA-E7 matured DCs have higher lytic efficiency compared to E7 stimulated alone for SiHa-HLA-A*02:01. Additionally, EDA-E7 induced unspecific T cell response, indicated by the lysis of wild type SiHa, was much lower than the lysis efficiency against SiHa-HLA-A*02:01 cells (19.1% vs. 27.1%) (Figure 7C).

## 4. Discussion

Cervical cancer is one of the main malignant tumors that endanger the health of women worldwide. The fact that 99% of cervical cancer patients were positive for HPV while type 16 HPV in Southwest of China is as high as 73.8% [3,4] makes the vaccination of cervical cancer via HPV possible. Compared to checkpoint inhibitor immunotherapy such as anti-PD-1 or anti-PD-L1 therapy, which relies largely on the expression of PD-L1 in cancer cells, vaccination with HPV antigen seems more promising. Indeed, a preventive vaccine against HPV has already shown great potential to prevent 90% occurrence of cervical cancer [22,23]. However, tumor immunosuppressive microenvironment including the recruitment of regulatory T cells and myeloid derived suppressor cells, as well as hypoxia, high interstitial fluid pressure, and physical barriers, make the immune response not efficient for therapeutic vaccinations for established tumor. EDA-E7 as a therapeutic vaccine has already been shown to eradicate large tumor in mice by repeated intratumor injection, which could possibly be because EDA could not just activate DCs through TLR4 signaling pathway, but also create a pro-inflammatory microenvironment in tumor as reported by other groups, and attract the infiltration of activated T cells [10]. In addition, in vitro generation of HPV vaccine is expensive and time consuming, as well as difficult to standardize each batch of product. Therefore, how to generate efficient therapeutic vaccine for HPV positive cervical cancer is of great interest.

The spliced exon encoding the type III repeat extra domain A (EDA) from fibronectin could target antigens to DCs and induce maturation through TLR4 [13]. Furthermore, a mouse-derived EDA-E7 recombinant fused protein has been shown to induce maturation of DCs and was able to eradicate well-established tumors expressing HPVE7 protein in mouse system [14]. In this study, we generated human derived EDA-HPVE7 fused protein and confirmed that this recombinant protein maintains the pro-inflammatory property of the EDA domain as well as to induce the maturation of DCs through binding of HPV16E7. From Figure 2B we can see that EDA-E7 could bind to DCs and upregulate antigen presenting molecules and costimulatory molecules. Furthermore, 500 nM concentration of EDA-E7 was found to achieve the best activation of DCs (Figure 3). TLR agonist was reported to work in synergy with EDA-E7 to eradicate established tumors through induction of pro-inflammatory cytokines such as TNFα and IL12. In this study, we also found that the recombinant protein EDA-E7 and TLR agonist could work in synergy to promote the secretion of cytokines from DCs to achieve functional maturity. Since it is difficult to use a humanized mouse model to mimic human immune system for eradicating established tumor in vivo, we used in vitro experiment to evaluate whether the DCs could induce antigen specific T cells. From a T cell activation marker as well as target killing experiments, we can conclude that naïve T cells were indeed activated after incubation with EDA-E7 and TLR agonist matured DCs. TCR-coupled single cell RNA sequencing indicated TCR clonal selection and amplification of T cells. In vitro T cell cytotoxic experiment indicated that EDA-E7 could induce HLA restricted antigen specific T cells response against HPV infected cervical cancer cell (Figure 7). In the future, we will continue to use humanized mouse models to verify the effect of EDA-E7 in inducing antigen specific T cells and eradication efficiency of HPV infected cervical cancer in vivo.

In conclusion, we synthesized a human origin fusion protein EDA-E7, which could induce maturation of human DCs and activate anti-HPV infected cervical cancer immune responses in vitro. Moreover, combined use with a TLR agonist such as poly (I:C) will achieve better maturation of DCs and T cell activation. Our study found that EDA-E7 could activate human T cells to kill human cervical tumor cells arising from HPV infection, which indicates it is worthwhile to investigate these observations further in humanized mice.

## 5. Conclusions

In this study, we successfully synthesized fusion protein EDA-E7 from human fibronectin and human HPVE7, and found that EDA-E7 could be efficiently captured by human PBMC derived dendritic cells (DCs) in vitro and induce DCs maturation. Importantly, this effect can work in synergy with the TLR ligand anti-CD40 agonist, polyinosinic-polycytidylic acid [poly (I:C)], R848, and CpG2216. EDA-E7 matured DCs could activate T cells and trigger anti-tumor response in vitro. Single RNA sequencing and T cell target killing assay confirmed the activation of T cells by EDA-E7 matured DCs. These results demonstrated that therapeutic vaccination with EDA-E7 fusion protein is effective in human cervical carcinoma treatment.

## Figures and Tables

**Table 1:** Primers for over-lap PCR.

Primer Number	Primer Name	Primer Sequence (5′-3′)
1	E7(1-29aa)up	GGAATTCCATATGCATGGAGATACACCTAC
2	E7(1-29aa)down	CCTTTAGGGCGATCAATGTTATTTAATTGCTCATAACAGT
3	EDA up	AACATTGATCGCCCTAAAGG
4	EDA down	TGTGGACTGGGTTCCAATCA
5	E7(43-98aa)up	TGATTGGAACCCAGTCCACAGGACAAGCAGAACCGGACAG
6	E7(43-98aa)down	ATAAGAATGCGGCCGCTGGTTTCTGAGAACAGATGG
7	E7(1-29aa)down-2	ATTTAATTGCTCATAACAGT
8	E7(43-98aa)up-2	TCTCTACTGTTATGAGCAATTAAATGGACAAGCAGAACCGGACAG

Wavy line indicates the over-lap sequence. Straight line indicates Ndel or NotI enzyme restriction site.

## Data Availability

The data presented in this study are available on request from the corresponding author.
